# Genome-wide analysis reveals the ancient and recent admixture history of East African Shorthorn Zebu from Western Kenya

**DOI:** 10.1038/hdy.2014.31

**Published:** 2014-04-16

**Authors:** M N Mbole-Kariuki, T Sonstegard, A Orth, S M Thumbi, B M de C Bronsvoort, H Kiara, P Toye, I Conradie, A Jennings, K Coetzer, M E J Woolhouse, O Hanotte, M Tapio

**Affiliations:** 1 Ecology and Evolution, School of Life Sciences, University of Nottingham, Nottingham, UK; 2 United States Department of Agriculture, Agriculture, Agricultural Research Services, Bovine Functional Genomics Laboratory, Beltsville, MD, USA; 3 International Livestock Research Institute, Nairobi, Kenya; 4 Centre for Immunity, Infection and Evolution, Ashworth Laboratories, University of Edinburgh, Edinburgh, UK; 5 The Roslin Institute, Easter Bush, University of Edinburgh, Edinburgh, UK; 6 Department of Veterinary Tropical Diseases, Faculty of Veterinary Science, University of Pretoria, Onderstepoort, South Africa; 7 MTT FI-31600, Jokioinen, Finland

## Abstract

The Kenyan East African zebu cattle are valuable and widely used genetic resources. Previous studies using microsatellite loci revealed the complex history of these populations with the presence of taurine and zebu genetic backgrounds. Here, we estimate at genome-wide level the genetic composition and population structure of the East African Shorthorn Zebu (EASZ) of western Kenya. A total of 548 EASZ from 20 sub-locations were genotyped using the Illumina BovineSNP50 v. 1 beadchip. STRUCTURE analysis reveals admixture with Asian zebu, African and European taurine cattle. The EASZ were separated into three categories: substantial (⩾12.5%), moderate (1.56%<X<12.5%) and non-introgressed (⩽1.56%) according to the European taurine genetic proportion. The non-European taurine introgressed animals (*n*=425) show an unfluctuating zebu and taurine ancestry of 0.84±0.009 s.d. and 0.16±0.009 s.d., respectively, with significant differences in African taurine (AT) and Asian zebu backgrounds across chromosomes (*P*<0.0001). In contrast, no such differences are observed for the European taurine ancestry (*P*=0.1357). Excluding European introgressed animals, low and nonsignificant genetic differentiation and isolation by distance are observed among sub-locations (*F*_st_=0.0033, *P*=0.09; *r*=0.155, *P*=0.07). Following a short population expansion, a major reduction in effective population size (*N*_e_) is observed from approximately 240 years ago to present time. Our results support ancient zebu × AT admixture in the EASZ population, subsequently shaped by selection and/or genetic drift, followed by a more recent exotic European cattle introgression.

## Introduction

The East African cattle group is a valuable genetic resource with a complex origin. The first African cattle were of taurine type *Bos taurus* (Gifford-Gonzalez and Hanotte, 2011). According to latest mitochondrial DNA results they originated from the geographic center of cattle domestication in the Near East and separated from the other taurine types approximately 7000 years ago (Bonfiglio *et al.*, 2012). These taurine cattle entered Africa through its North-Eastern part via present day Egypt (Epstein, 1971; Blench and MacDonald, 2000; Gifford-Gonzalez and Hanotte, 2011). Zebu cattle (*Bos indicus*) originated in the Indian subcontinent and migrated into Africa more recently (Gifford-Gonzalez and Hanotte, 2011). The earliest undisputed evidences of zebu cattle dated from the first mid-millennium AD (Gifford-Gonzalez and Hanotte, 2011). They may have subsequently penetrated Africa in two waves (Hanotte *et al.*, 2002), with the second wave possibly facilitated by the rinderpest epidemic (Blench, 1993; Paynes and Hodges, 1997). Contemporary cattle from the eastern part of Africa are predominantly phenotypically classified as zebu (Rege and Tawah, 1999; DAGRIS, 2007). As to whether or not the African aurochs *B. primigenius africanus* (now extinct) contributed to the genetic stock of African domestic cattle remains unknown (Gifford-Gonzalez and Hanotte, 2011).

Currently, Africa is home to over 150 recognized cattle breeds that comprise of a mosaic of zebu, taurine and crossbreeds (indicine and taurine), the latter sometimes referred to as sanga (Rege and Tawah, 1999). In Kenya, owing to the effect of tribal boundaries and socioeconomic cultures, different strains of the East African Shorthorn Zebu (EASZ) are recognized (Rege, 1999). These include the Kavirondo zebu reared by the Luo and Luhya communities, and the Teso zebu reared by the Teso community who mainly inhabit western Kenya (DAGRIS, 2007). Genetic studies carried out using microsatellite markers (Rege *et al.*, 2001) show that Kenyan zebu populations are zebu–taurine hybrids with a major zebu genetic component. Further studies with Y-chromosomal markers (Hanotte *et al.*, 2000) and mitochondrial DNA markers (Bradley *et al.*, 1996) are in agreement with a male-mediated zebu introgression of the taurine animals.

In more recent times, a wave of ‘exotic' taurine cattle introductions has set off following governmental and international livestock development agendas aimed at improving livestock productivity (Hanotte *et al.*, 2002). These cattle are of European taurine genetic backgrounds and include breeds such as Holstein–Friesian and Jersey as reported in Kenya (Weir *et al.*, 2009) and Ethiopia (Haile *et al.*, 2011). The rapidly changing socioeconomic and cultural environment of rural farming is favoring these introductions. This shift of focus is to a perceived economically beneficial animal as opposed to an ecologically fit one, leading to active breed replacement or crossbreeding programs within Eastern Africa, particularly in the highlands and peri-urban areas (Bebe *et al.*, 2003).

Today, indigenous zebu cattle are the commonest cattle type across most parts of Eastern Africa (Rege and Tawah, 1999). Their wide distribution is a possible consequence of their environmental adaptive traits not found in exotic taurine breeds. These include resistance or tolerance to tropical diseases and their vectors (Latif *et al.*, 1991a, 1991b; Mattioli *et al.*, 1993; Lawrence *et al.*, 1996; Wambura *et al.*, 1998; Hanotte *et al.*, 2003), survival on poor quality forages/pastures (Bonsma, 1973; Turner, 1980) and thermal stress tolerance (Carvalho *et al.*, 1995; Hammond *et al.*, 1996; Gaughan *et al.*, 1999; Hansen, 2004). In addition, African zebu cattle are still a reliable source of draught power (Rege *et al.*, 2001).

African taurine (AT) are on the verge of extinction in East Africa (Rege, 1999), but their environmental genetic adaptation (for example, infectious disease tolerance) may have survived in East African zebu and their crossbreeds. However, despite being well adapted to the harsh tropical environment, African zebu and taurine cattle remain poor producers in comparison with exotic breeds raised in the temperate environments (Rege *et al.*, 2001). A medium to long-term solution for sustainable improvement of productivity in the tropics may be to combine ecologically adaptive zebu traits and economically important exotic cattle traits in crossbreed animals.

The recent availability of genome-wide scan tools are offering new opportunities for genetic characterization (Gautier *et al.*, 2009, 2010; McTavish *et al.*, 2013), genome-wide association studies (Van Tassell *et al.*, 2008; Matukumalli *et al.*, 2009; Settles *et al.*, 2009; Pant *et al.*, 2010), the detection of signatures of selection for productivity (Barendse *et al.*, 2009) and genomic evaluation (Wiggans *et al.*, 2008, 2010) of cattle populations. Most of these studies have been carried out predominantly on European dairy and beef cattle breeds (The Bovine HapMap Consortium, 2009; Melka and Schenkel, 2012) with the exception of Gautier *et al.* (2009) and Gautier and Naves (2011) who worked on West African and Caribbean Creole cattle, respectively.

In this study, we characterize at genome-wide level the genetic diversity and architecture of 548 EASZ, an indigenous cattle population from Kenya. We present evidences for ancient zebu × taurine admixture, population bottleneck and expansion as well as the presence of recent and ongoing exotic taurine introgression. No significant genetic differentiation in non-European introgressed animals is observed across the studied sites. Moreover, our study further provides insight in the usefulness and limitations of low-density single-nucleotide polymorphisms (SNPs) chips toward understanding the genomic architecture and history of indigenous African tropical cattle.

## Materials and methods

### Study cohort and sampling site

A total of 548 calves were sampled from 20 different randomly selected sub-locations that traverse four distinct ecological zones in Western and Nyanza provinces of Kenya (Supplementary Figure S1). Upon recruitment, blood samples were drawn from the jugular vein using a 10 ml sterile syringe. Five ml of blood was mixed in sodium EDTA tubes in a 1:1 ratio with ‘magic buffer' (which acted as an anti-coagulant, anti-fungus, anti-bacterial and preservative—Biogen Diagnostica, Villaviciosa De Odon, Spain). The tubes were labeled with their respective bar-coded tags before being stored at 4 °C at the International Livestock Research Institute—ILRI (Nairobi, Kenya) biobank. DNA was extracted using the Nucleon Genomic DNA extraction kit (Tepnel Life Sciences, Manchester, UK).

### Genotyping and quality control

The Illumina BovineSNP50 v. 1 beadchip (Illumina Inc., San Diego, CA, USA) includes 56 947 SNPs comprising 54 436 autosomal SNPs, 1341X chromosome SNPs and 1170 unmapped SNPs (the mapping of the genomic positions was done using the University of Maryland genome assembly v 3.0; www.cbcb.umd.edu/research/bos_taurus_assembly.shtml). Genotyping of the 548 calves was carried out at the USDA-ARS bovine functional (Beltsville, MD, USA) and GeneSeek (http://www.neogen.com/geneseek/) laboratories. An additional 158 reference animals representing two European taurine breeds (Holstein *n*=64 and Jersey *n*=28), one AT breed (N'dama *n*=25), one Asian zebu breed (Nelore *n*=21) and one East African admixed breed (Sheko *n*=20, Ethiopia) breeds were drawn from the Bovine HapMap Consortium (2009).

Quality control was carried out using *GenABEL* program (Aulchenko *et al.*, 2007) in R (R Development Core Team, 2009). The *check.marker* function was used to prune individual calves that failed to pass the inclusion criterion of successful genotypes per calf of >90% and an average identical by state threshold of >90% (excluding both animals).

Only mapped autosomal SNPs (*n*=54 436) were screened. Autosomal SNPs with call rates of <90% were excluded. Unless stated, no minor allele frequency threshold (to avoid exclusion of SNPs that maybe informative in a single breed or population) and Hardy and Weinberg Equilibrium (as introgression may result in loci not in Hardy and Weinberg equilibrium) criteria, were applied. A total of 6151 SNPs failed the inclusion criterion, leaving a total 48 285 SNP for analysis. A random subset of 45 000 SNPs was used for STRUCTURE, principal component analysis, genetic relatedness and genetic differentiation analyses. No random sampling of SNPs was performed for linkage disequilibrium (LD) and effective population size calculations. Genotyping data has been deposited in Dryad (Murray *et al.*, 2013a).

### Admixture analysis

The extent of admixture and the origin of the different genetic proportions were investigated, using a Bayesian clustering method implemented in the STRUCTURE program (Pritchard *et al.*, 2000, 2007). Five independent replicates of an admixed model with independent allele frequencies were run for a burn-in period of 50 000 iterations and 100 000 Markov Chain Monte Carlo steps for *K*=1 to *K*=7. The mean output files from CLUster Matching and Permutation Program—*CLUMPP* v 1.1.2 (Jackobsson and Rosenberg, 2007) were used as input files to graphically display the population structure barplots using the *barplot* function in R (R Development Core Team, 2009). The population structure analyses using 45K markers were run on three calf sample sets: (i) full study population (*n*=548) and reference breeds (*n*=158) totaling 706 animals (data set 1), (ii) non-European introgressed calves (*n*=425) and reference breeds (*n*=158) giving a total of 583 animals (data set 2) and (iii) non-European introgressed calves (*n*=425) (data set 3). The Ward clustering method (Ward, 1963) using the *hclust* function in R (R Development Core Team, 2009) was used to identity discontinuities in the distribution of the European taurine ancestry within data set 1. Multiple methods were used to evaluate the optimal number of genome backgrounds within the study population (Falush *et al.*, 2003; Evanno *et al.*, 2005; Pritchard *et al.*, 2007).

### Principal component analysis

Principal component analyses were applied on the full study population and reference breeds (data set 1) and the non-European introgressed calves (data set 3) using *adegenet* v 1.3.1 (Jombart, 2008) to read the data files into R and *ade4* genetic package to calculate the principal components and eigenvalues (Dray and Dufour, 2007). Both packages are found in R (R Development Core Team, 2009).

### Genetic relatedness and genetic differentiation

Global and pairwise *F*_st_ statistics were calculated using the R-based *hierfstat* 0.04–6 package (Goudet, 2005). The assessment of the influence of physical geographic distance on genetic distance between calf pairs within and between the sub-locations (data set 3) was tested with the Mantel test. The analysis was carried out with *adegenet* v 1.3.1 (Jombart, 2008) genetic package in R (R Development Core Team, 2009). The geographic coordinates were converted to kilometers using conversion units based on the World Geodetic System 1984 (WGS84) spheroid. The functions *dist* and *mantel.randtest* were used to calculate the pairwise geographic distances and the Mantel test statistic, respectively. The pairwise rescaled *F*_st_ estimates (*F*_st_/(1−*F*_st_) were used for the estimation of genetic differentiation between sub-locations in relation to geographic distance (Rousset, 1997).

### LD and effective population size in pure EASZ

Pairwise LD, measured as squared correlation coefficient *r*^2^ (Hill and Robertson, 1968), was calculated in *GenABEL* (Aulchenko *et al.*, 2007) for the non-introgressed European calves (data set 3). The decrease of LD as a function of distance between markers was evaluated for pairs up to 4 Mb apart using markers with minor allele frequency above 0.01. The expected value was predicted with Loess local regression of second degree using the 5% of the data closest to the estimation point in the local regression as implemented in R (R Development Core Team, 2009). For estimating the effective population size, 11 bins (<0.1, 0.1–0.2, 0.2–0.3, 0.3–0.5, 0.5–1.0, 1.0–1.5, 1.5–2.0, 2.0–2.5, 2.5–3.0, 3.0–3.5 and >3.5 Mb) were used, and the mean for each bin used to obtain the expected *r*^2^.

Estimation of the ancestral effective population size was calculated using the Weir and Hill (1980) adjusted formula, *E* (*r*^2^)=[1/(1+4*N*_e_c)]+(1/*n*). Where *E* (*r*^2^) is the expected LD, *N*_e_ is the effective population size, *c* is the recombination frequency, *n* is the chromosome size or twice the sample size, *c* was calculated using the Haldane's mapping function (Haldane, 1919), with the average marker distance between adjacent SNPs expressed in Morgans, assuming 1 Mb=1 cM (de Roos *et al.*, 2008). The estimate for a bin relates to the *N*_e_ for the generation *t*=1/2*c* (Hayes *et al.*, 2003) counted backwards from the genotyped generation. A generation length of 6 years was assumed (Mahadevan, 1955).

### Performance and ascertainment bias of the Illumina BovineSNP50 v.1 beadchip in Kenyan EASZ

To assess the Illumina BovineSNP50 v.1 beadchip performance as a tool of estimating zebu and taurine admixture proportions. STRUCTURE analyses at *K*=3 were performed using data sets of randomly selected sets of SNPs (*n*=5000, 10 000, 15 000, 25 000, 35 000 and 45 000) drawn from the cleaned data set of 48 285 markers. These particular analyses were run using the moderate and substantial calf categories consisting of 123 calves and 158 animals representing the five reference breeds (total number 281). Ancestral genome proportions were generated from the STRUCTURE runs and used in the subsequent linear correlation analyses carried out between markers sets in R (R Development Core Team, 2009).

## Results

Out of 48 285 autosomal SNPs remaining in the data set following quality control, 11 269 markers were monomorphic across the EASZ population. The mean observed heterozygozity (*H*o) within EASZ calves (*n*=548) and reference breeds are indicated in Table 1. EASZ population shows an average *H*o of 0.25±0.02 s.d. with no significant differences across sub-locations (*P*>0.001).

**Figure 1:**
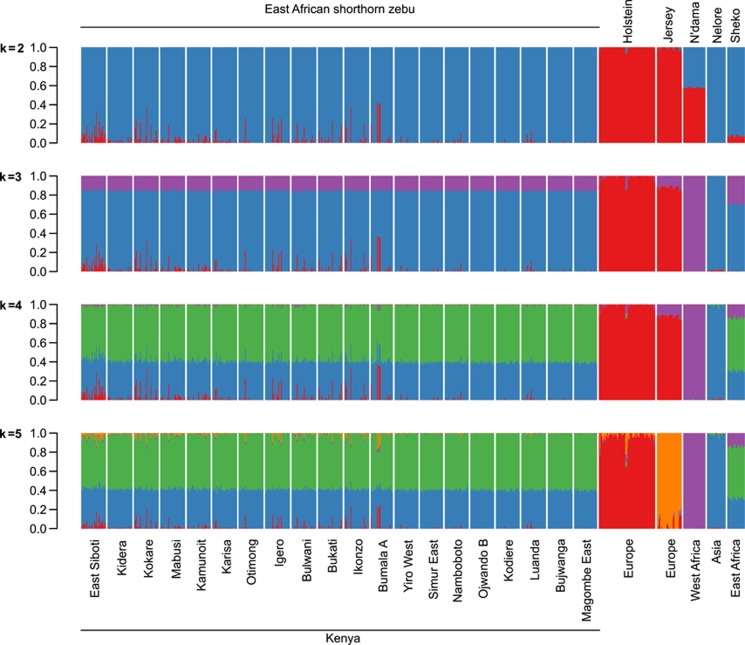
STRUCTURE bar plots of genetic membership proportions (*K*=2 to *K*=5). Each animal is represented by a vertical line divided into *K* colors. Breed names and locations are indicated at the top and bottom of the bar plots, respectively.

STRUCTURE runs from *K*=2 to *K*=5 using 45 000 random SNPs are presented in Figure 1. Applying the Evanno *et al.* (2005) method suggests *K*=2 as the optimal partition (Supplementary Figure S2), which is the uppermost relevant hierarchy reflecting the taurine and indicine cattle split. A clear but less drastic improvement in the fit of the model is visible by increasing *K* to 3, revealing previously documented findings that highlight a further taurine split to African and European taurines (The Bovine HapMap Consortium, 2009). The increase from 3 to 4 genetic clusters is minimal and does not lead to a new individual breed cluster. However, a notable improvement is observed when increasing *K* to 5, revealing a finer resolution separating the two European taurine breeds (Supplementary Figure S2). Above *K*=5, the increase in goodness of fit with larger *K* values are only incremental, suggesting that they do not reveal significant phylogenetic structure (Falush *et al.*, 2003, Prichard *et al.*, 2007).

**Figure 2:**
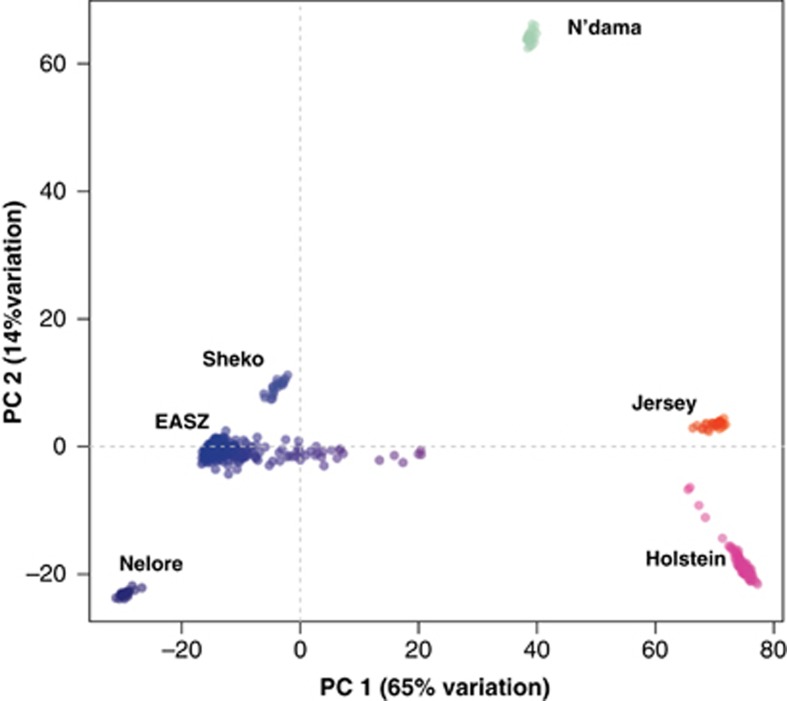
Principal component analysis plot of EASZ and reference breeds. PC1 and PC2 explain 65% and 14% of the total variance, respectively.

STRUCTURE results of *K*=3 are in agreement with prior information about the main genetic architecture of the cattle on the African continent being of three different ancestries using microsatellite loci; Asian zebu *B. indicus*, AT *B. taurus* and European taurine *B. taurus* (Hanotte *et al.*, 2002; The Bovine HapMap Consortium, 2009). Of interest, a subset of the EASZ calves has European taurine ancestry, while the other East African cattle breed studied, the Sheko, shows no European taurine introgression (Supplementary Table S1). In addition, at *K*=3, the Jersey breed presents a shared genetic background (mean ancestral proportion −0.12, Supplementary Table S1) with the N'dama breed. However, this is not observed at *K*=5 (Figure 1). Possible AT membership is also observed in some Holstein–Friesian animals up to a proportion of 0.10–0.11 (*n*=3) (Supplementary Table S1). Principal component analysis on the same data set shows that PC1, explaining 65% of the variation, separates the indicine and taurine breeds; whereas PC2, explaining 14% of the variation, separates the AT breed (represented here by the N'dama of West Africa) from the European breeds (Figure 2). For *K*=4, the proportion of European taurine in EASZ remains the same, but not the inferred ancestral proportion of zebu and AT background (Figure 1). In addition, there is hardly any genetic background shared between the EASZ and the West African cattle (N'dama). However, a substantial proportion of the EASZ genome remains shared with the Nelore (Asian zebu). The largest proportion of genome ancestry present in EASZ is now nearly unique to the EASZ and Sheko with only traces of it found within the Nelore (Figure 1). *K*=5 divides the inferred European ancestry between the two European breeds (Jersey and Holstein–Friesian) (Figure 1).

**Figure 3:**
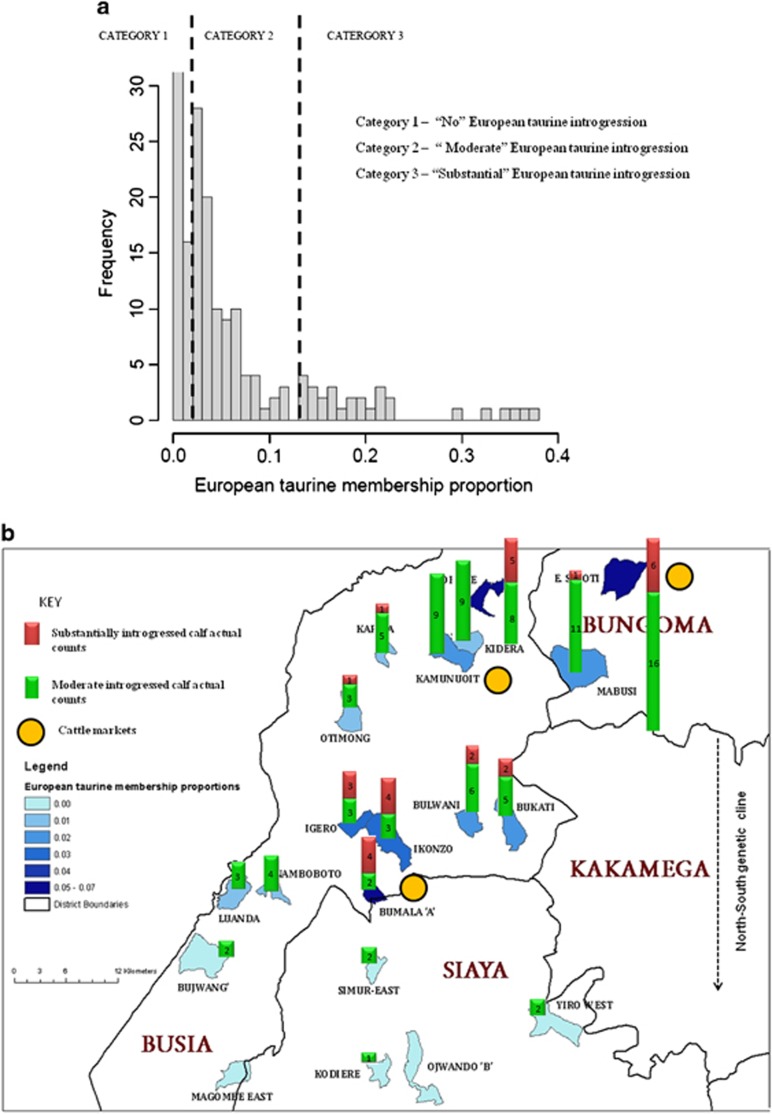
(**a**) Histogram representing the frequency of calves with varying levels of European ancestry. Three categories of European taurine introgression of data set 1 were defined using the Ward algorithm. (**b**) Geospatial distribution of the substantial European taurine (⩾12.5%) and moderate European taurine (1.56%<X<12.5%) categories. It indicates a genetic cline and two hotspots of substantial European introgression (⩾12.5%) found within close proximity of animal markets.

Using the Ward clustering method (Ward, 1963), we further analyzed the proportion of European taurine background in the EASZ (Figure 3a) based on the *K*=3 model. Based on three observed clusters, we defined three categories of calves: calves with ⩾12.5% European taurine background (category 1 representing animals with ‘substantial' European introgression *n*=29), calves with between 1.56 and 12.5% European taurine introgression (category 2 representing the ‘moderate' European taurine introgressed sample set *n*=94) and calves with ⩽1.56% European taurine background (category 3 representing the ‘non-European' introgressed sample set *n*=425). The 123 introgressed calves, representing the moderate and substantial categories, are found within 12 sub-locations in the northern and central regions of the study area (Supplementary Table S1). A geospatial analysis of the substantial category reveals two main hotspots of European taurine introgression (Figure 3b), whereas the moderate category shows a north to south decrease of European taurine introgression (*r*=0.82, *P*<0.0001; Figure 3b).

STRUCTURE analysis (*K*=3) of data set 2 reveals a homogenous admixed EASZ population (Supplementary Figure S3) with average genetic proportions of 0.84±0.009 s.d. and 0.16±0.009 s.d. of AT and Asian zebu ancestries, respectively. Significant genome-wide difference of AT or zebu ancestry across calves (*P*<0.0001) was also observed.

Interestingly, the chromosome-wise analyses reveal that some of the ‘non-introgressed' calves (Supplementary Figure S4a) have moderate proportion of European taurine ancestry on a small subset of chromosomes, similarly within the Sheko population. There are highly significant differences (*P*<0.0001, Supplementary Figure S4b and Supplementary Table S2) among chromosomes in the amount of AT ancestry in both introgressed and non-introgressed EASZ calves. However, for the same genetic ancestry (AT) in the Sheko population, no significant differences were observed (*P*>0.05; Supplementary Table S2). For the European taurine and Asian zebu ancestries, highly significant differences between chromosomes are observed in the non-introgressed calves' cohort unlike in the Sheko population (*P*>0.05; Supplementary Table S2), as illustrated in Supplementary Figures S4a and c, respectively. Differences among calves are highly significant for European taurine ancestry in both introgressed and non-introgressed calves, as well as for Asian zebu ancestry in introgressed calves (Supplementary Figures S4a and c). In contrast, there are no differences in the AT ancestry among all the calves' cohorts and Sheko population (Supplementary Figure S4b).

Strong positive correlations for the AT and Asian zebu ancestry between EASZ and Sheko are observed across chromosomes (*r*=0.89, *P*<0.0001 and *r*=0.94, *P*<0.0001). Considering only EASZ calves with moderate and substantial European taurine introgression, no significant difference is observed in European taurine ancestry proportion across chromosomes (*P*=0.136).

Data set 3 allows addressing the possible presence of sub-structuring within the non-European taurine introgressed EASZ population. PC1 and PC2 explain 4% and 3% of the percentage of the total variance, respectively (Supplementary Figure S5a). Three genetic clusters are observed of which the largest includes 415 calves, while the second and third clusters include 4 and 6 calves from Bukati and Luanda sub-locations, respectively (Supplementary Figure S5a). This result is further illustrated with the STRUCTURE results (*K*=3), which distinguishes the same calves (Supplementary Figure S5b).

No genetic differentiation is observed between sub-locations (*F*_st_=0.0033, *P*=0.09) after removing the moderate and substantial European introgressed calves. Mantel test between pairwise sub-locations genetic differentiation (calculated as (*F*_st_/(1−*F*_st_)) and geographic distances was not significant (*r*=0.155, *P*=0.07) for the pure calves category (Supplementary Figure S6).

The extent of LD over genome regions is related to effective population size. In the pure EASZ population, the LD decreased quickly (Supplementary Figure S7). The expected value was estimated with Loess curve based on a total of 2 368 859 comparisons between markers. At the minimum distance, the expected *r*^2^ is 0.32. It decreases to 0.2 at 55.4 kb, to 0.1 at 200.3 kb and to 0.05 at 486.0 kb distance. LD begins to asymptote at the background level of *r*^2^=0.013 around 2 MB. The LD results were used to estimate the effective population size.

**Figure 4:**
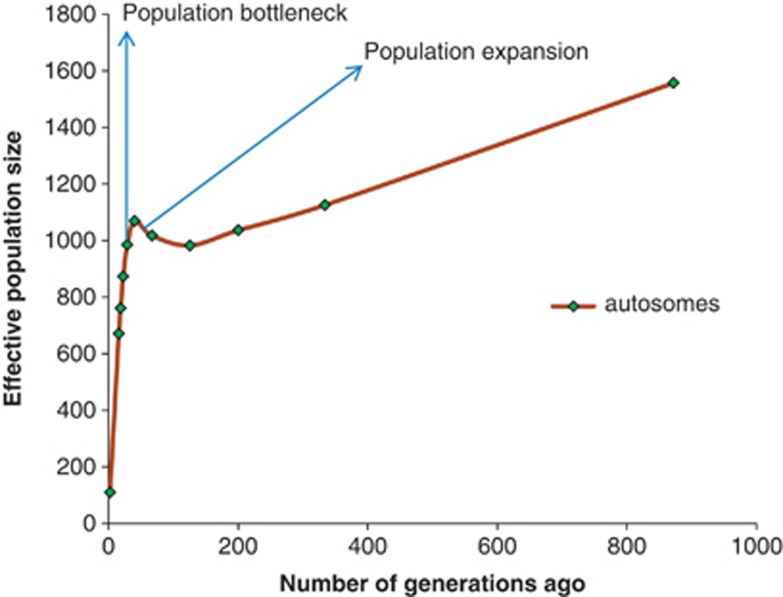
General trend of the average effective population size variation against past generations.

The effective population size is characterized by a population decline over time (Figure 4). However, a short stint of increasing population size, starting around 126 generations ago (∼756 years ago), is observed before a drastic population decrease starting around 40 generations ago (∼240 years ago) and continuing to present day (Figure 4).

We assessed the effect of the number of markers for the estimation of genome ancestry in our population assuming three ancestral populations (indigenous AT, European taurine and Asian zebu) and using only calves with moderate and substantial European taurine introgression. We use linear correlation between sets of randomly selected number of markers using STRUCTURE's output of inferred genetic membership proportions at *K*=3. The analyses indicate that the estimation of the proportion of genome ancestry varied with the number of markers included, and that it depends on the genetic background being estimated (Supplementary Figures S8a–c). As expected, the more markers the better the correlation. More particularly, a correlation coefficient of *r*=0.99 (*P*<0.0001) was obtained for the 15–25K pairwise marker comparison for the European background (Supplementary Figure S8a), and for the 25–35K pairwise marker comparison for the Asian zebu ancestry (Supplementary Figure S8b). However, for the estimation of the AT ancestral background a correlation coefficient of only *r*=0.92 (*P*<0.0001) was obtained for the 35–45K pairwise marker comparisons (Supplementary Figure S8c).

Pruning neighboring markers in strong LD (*r*^2^ over 0.1–0.5) has been suggested as a way to reduce data redundancy in STRUCTURE analysis and ascertainment bias in the estimation of diversity. Approximately 44% of the neighboring marker pairs linkage were above the lower limit (*r*^2^=0.1) and only 11% above the higher limit (*r*^2^=0.5) within the non-introgressed calves cohort. The proportion of marker pairs in LD approximates the genome coverage of the chip, for example, in mapping applications. Taking into account the marker gaps, these proportions equal to coverage of 40 and 8%, with the respective *r*^2^ limits. The median for neighboring marker pairs was 0.075. Therefore, no LD-based pruning was undertaken.

## Discussion

This study aimed at unraveling the population history and genetic structure of an indigenous population of EASZ of western Kenya at genome-wide level. At *K*=3, STRUCTURE analysis agrees with the principal component analysis dimension results indicating the presence of three genetic backgrounds. This is not surprising given our current understanding of the origin and history of these populations and it is in agreement with previous finding using microsatellite loci (Hanotte *et al.*, 2002). Moreover, using 45 000 randomly selected autosomal genome-wide SNP markers, we are able to unravel finer details of the extent of genome admixture (AT, European taurine and Asian zebu) within the studied populations (Figures 1 and 2). Assessment of the level of inferred ancestral proportion difference between animals (two EASZ calf cohorts and the Sheko breed) and autosomes (Supplementary Figures S4a–c) is also presented.

Our results support ancient zebu × AT admixture in the EASZ population, subsequently shaped by selection and/or genetic drift, followed by a more recent exotic European cattle introgression. Indeed, we do observe very little variation among animals for the inferred zebu and AT ancestral proportions, at the contrary of the inferred European taurine background. It indicates that the AT and zebu genome ancestries have had time to ‘diffuse' homogenously among the calves of our study population, while the level of European taurine introgression, of more recent origin, still needs to reach an equilibrium (Figure 1). Interestingly, we observe differences in zebu or AT ancestry proportions among chromosomes, differences shared between the EASZ and the Sheko as revealed by the correlation analyses. It suggests at least a partial role of selection in shaping the genome architecture of present day indigenous East African cattle populations rather than only genetic drift. The EASZ and Sheko admixed (taurine × zebu) populations occupy to some extent similar agro-ecological environments in different geographical locations (DAGRIS, 2007). It remains, however, unknown which common selection pressures may have shaped the genome of these crossbreed populations. These effects may be attributed to environmental factors (for example, common infectious disease challenges) and/or may be the consequence of within genome selection pressures following the crossbreeding of cattle belonging to two distinct lineages that separated more than half a million years ago (Loftus *et al.*, 1994; MacHugh *et al.*, 1997). Worth mentioning here, is the detected presence so far of only taurine cattle mitochondrial DNA on the African continent even in populations phenotypically classified as zebu (Gifford-Gonzalez and Hanotte, 2011), is an observation compatible with the pattern of male-mediated zebu introgression into taurine animals or with a selection pressure in favor of taurine mitochondrial haplotypes.

We also assessed if the global genome admixture of the EASZ could be further partitioned to finer detail. In other words, whether the AT and Asian zebu inferred genetic ancestry in EASZ at *K*=3 may be further separated into distinct genome components that reflect in-depth details revealing the history of the breed. Previous studies indicate that through the Horn of Africa, the African continent likely witnessed two waves of zebu introductions and migrations (Hanotte *et al.*, 2002), which may have distinctively imprinted the genome of the EASZ. The presence of a unique shared genetic background at *K*=4 (Figure 1) between EASZ and Sheko, absent in both the Nelore and N'dama breeds, is compatible with a two wave zebu introgression pattern. It would be tempting to claim that this additional component may represent the first phase intermediate zebu–taurine hybrid, so-called sanga cattle (see Rege, 1999; Rege and Tawah, 1999), but in absence of an appropriate reference population such interpretation remains hypothetical. Indeed, the presence of a ‘unique' East African genetic background will also be compatible with an indigenous African ancestry (for example, from African wild aurochs introgression). The analysis of more African cattle populations, representative of a broader geographic area from the continent, as well as additional reference breeds drawn outside of the continent may further clarify this issue. Until then, we should favor the clearly interpretable three clusters model previously supported by several studies (for example, Hanotte *et al.*, 2002).

As mentioned above, the level of European taurine ancestry in EASZ was unevenly distributed among calves, but relatively similar among chromosomes (Figure 1 and Supplementary Figure S4a). There are several possible European taurine genetic sources in the studied area. These include concluded and ongoing dairy breed improvement programs that used/use exotic animals and semen. Also, animal markets in the studied area are stocked with crossbred animals. Although the overall average European ancestry was only 2% for the entire studied population, the proportions varied across the study area. The animals with substantial European ancestry (⩾12.5%), which is compatible with a European taurine introgression event three or less generations ago, were found close to major livestock markets (Figure 3b). Interestingly, the average proportions of European taurine (ranging between 4 and 7%) were higher in northern sub-locations such as Busia and Bungoma that participated actively in initiatives to develop the local dairy production systems (Baltenweck *et al.*, 2005). In the southern sub-locations found within Siaya district, little or no European taurine introgression was observed (⩽1.25% Figure 3b). We purposely avoided the sampling of first-generation crossbred animals during the study and therefore our data is not representative of the current impact of dairy sector development activities on the indigenous EASZ population. Even so, our study still highlights the trend of European taurine introgression into the EASZ population. An interesting related question is whether or not such exotic introgression is under selection? Commercial cattle originating from the temperate environment are known to be poorly adapted to most of the tropical agro-ecosystems (for example, Vordermeier *et al.*, 2012) and selection against exotic introgression will be expected. Our recent analysis of the same EASZ animals suggests that it is indeed the case with increased vulnerability to infectious diseases for the EASZ introgressed animals (Murray *et al.*, 2013b).

Excluding introgressed animals with exotic taurine, nonsignificant genetic differentiation was observed between sub-locations (Supplementary Figure S6). Low or absence of differentiation implies frequent exchanges and/or movements of animals across a geographical area (Tapio *et al.*, 2010; Dumasy *et al.*, 2012). This is compatible with movement of livestock, including breeding bulls across the studied area and active animal trade between sub-locations. It may also explain why we do observe in two sub-locations (Bukati and Luanda) a few calves with different indigenous ancestral proportion. The parentage from these calves could have included other indigenous breed(s) present near the studied area.

EASZ are believed to comprise of several sub-populations according to the farmer communities rearing them. These include the Kavirondo zebu reared by the Luo and Luhya communities, and the Teso zebu reared by the Teso community (DAGRIS, 2007). Berthouly *et al.* (2009) have shown that, in Vietnamese goat, genetic differentiation is greatly influenced by farmers' ethnicity and husbandry practices. There is no apparent evidence that this may be the case in our study population.

Decrease in LD as a function of distance between markers has been reported in several cattle population or breeds (for example, de Roos *et al.*, 2008; Gautier *et al.*, 2009; Flury *et al.*, 2010). The observed intermediate LD in EASZ, 0.05 <*r*^2^<0.2 (Supplementary Figure S7), reaches approximately as far in the genome as in other cattle populations (de Roos *et al.*, 2008; Gautier *et al.*, 2009; Flury *et al.*, 2010), but both the minimum and maximum *r*^2^ were lower than commonly observed. The background LD (the minimum value to which the mean LD asymptotes with increasing distance between markers) is approximately a quarter of those generally observed in cattle populations (de Roos *et al.*, 2008; Gautier *et al.*, 2009; Flury *et al.*, 2010; Supplementary Figure S7). It is similar to the expected value for a heavily stratified population (Gautier *et al.*, 2007). This may suggest that our population has been subdivided in the past, although today we do not observe genetic differentiation across the studied area.

There are several algorithms for inferring population size from LD and it remains unclear which method is the most appropriate one (for example, Corbin *et al.*, 2012). However, cattle LD estimates indicate that all these populations, which include Asian zebu, AT and European taurine breeds, have been shrinking over time (Gautier *et al.*, 2007; de Roos *et al.*, 2008; The Bovine HapMap Consortium, 2009; Flury *et al.*, 2010). The trend has been associated with domestication events, artificial selection for economic traits and breed formation (The Bovine HapMap Consortium, 2009). We do observe a similar declining trend in population size for the EASZ (Figure 4). However, in contrast to other cattle populations, an increase in effective population size is observed approximately 126 generations ago.

Historical cattle generation lengths, prior the intensive breeding system, are expected to be longer than 4 years (Kidd and Cavalli-Sforza, 1974). We assume a 6 years generation length in the EASZ as estimated in Red Sindhi cattle on the Indian subcontinent (Mahadevan, 1955). It is slightly shorter than 6.72 years estimated by Alim (1960) for Kenana zebu cattle in Sudan. With this assumption, the EASZ expansion would have begun approximately 126 generations or ∼750 years ago, which is the time when zebu cattle supposedly became common in the Rift Valley (Payne, 1970). We then observe a drastic decrease in effective population size starting around 40 generation or 240 years ago up to present time (Figure 4). During this time span (∼240–750 years ago), three exceptionally favorable climatic periods with relatively shorter dry spells were experienced in Eastern Africa (Verschuren *et al.*, 2000). The cattle population seemed to thrive during these favorable seasons but drastically shrunk during the subsequent Lapanarat–Mahlatule drought that was characterized by a sequel of severe droughts and political upheavals (Verschuren *et al.*, 2000). It is also well documented that East African cattle were decimated following the rinderpest epidemic at the end of the nineteenth century (Blench, 1993; Paynes and Hodges, 1997). Our data suggests that the decline in cattle population in the region had already started before the disease outbreaks.

The BovineSNP50 v. 1 beadchip used in this study allowed us to estimate the ancestry proportions of the three main cattle lineages within the EASZ. The largest proportion of the markers included on this beadchip was selected for informativeness in European taurine breeds (Matukumalli *et al.*, 2009), and not surprisingly, a small number of these markers are sufficient for the estimation of the European ancestral proportion (Supplementary Figure S8a). This ascertainment bias makes the SNP chip particularly suitable for the detection of European taurine introgression in African native cattle populations. In contrast, more markers were required for accurate estimation of the zebu (Supplementary Figure S8b) and more particularly the AT ancestries (Supplementary Figure S8c). Owing to this technical shortcoming, the taurine ancestral estimates of our study population may not be fully conclusive. The current availability of a BovineHD genotyping bead chip (Illumina) offers an opportunity to clarify this issue. In addition, we show here that the neighboring markers in the BovineSNP50 v. 1 beadchip are not typically in strong LD, and the chip is informative only for approximately half of the EASZ genome, making the chip an incomplete tool for genome-wide association study, detection of signatures of selection and genomic selection.

The history of East African cattle is complex. It was closely intermingled with the history of local human communities (Hanotte *et al.*, 2002). Undoubtedly, the need to adapt to the harsh tropical environments of the area must have shaped the present day East African cattle genomes. This article presents valuable insights toward better understanding the genetic landscape, genetic affinities and demographic history of an African indigenous cattle breed as well as highlights the urgent need to implement crucial management strategies pertinent in the population's sustenance. The expected ‘*Livestock Revolution*' (Delgado *et al.*, 1999) describes a growing demand for animal products especially in developing countries. It presents a unique opportunity to harness the African indigenous livestock productivity potential by re-defining current breeding strategies aimed at obtaining both productive and resilient animals, while concurrently preserving the rich genetic variability of these populations. In-depth characterization of the genome of these indigenous African breeds is an essential step toward achieving these ultimate goals.

## Data archiving

Data available from the Dryad Digital Repository: doi:10.5061/dryad.bc598.

## Figures and Tables

**Table 1:** Heterozygozity estimates of EASZ and four reference breeds

*Breed*	*Observed heterozygozity* (H_*o*_)
EASZ	0.25±0.02 s.d.
Holstein–Friesian	0.33±0.01 s.d.
Jersey	0.25±0.03 s.d.
N'dama	0.17±0.08 s.d.
Ethiopian Sheko	0.26±0.003 s.d.

Abbreviation: EASZ, East African Shorthorn Zebu.
