# Supplementary material for: Genome-wide analysis reveals the ancient and recent admixture history of East African Shorthorn Zebu from Western Kenya
Source: Heredity (Edinb). 2014 Apr 16;113(4):297–305. doi: 10.1038/hdy.2014.31 (PMC4181064; doi:10.1038/hdy.2014.31)
Supplement: Supplementary Figure 1 [file hdy201431x1.pdf]

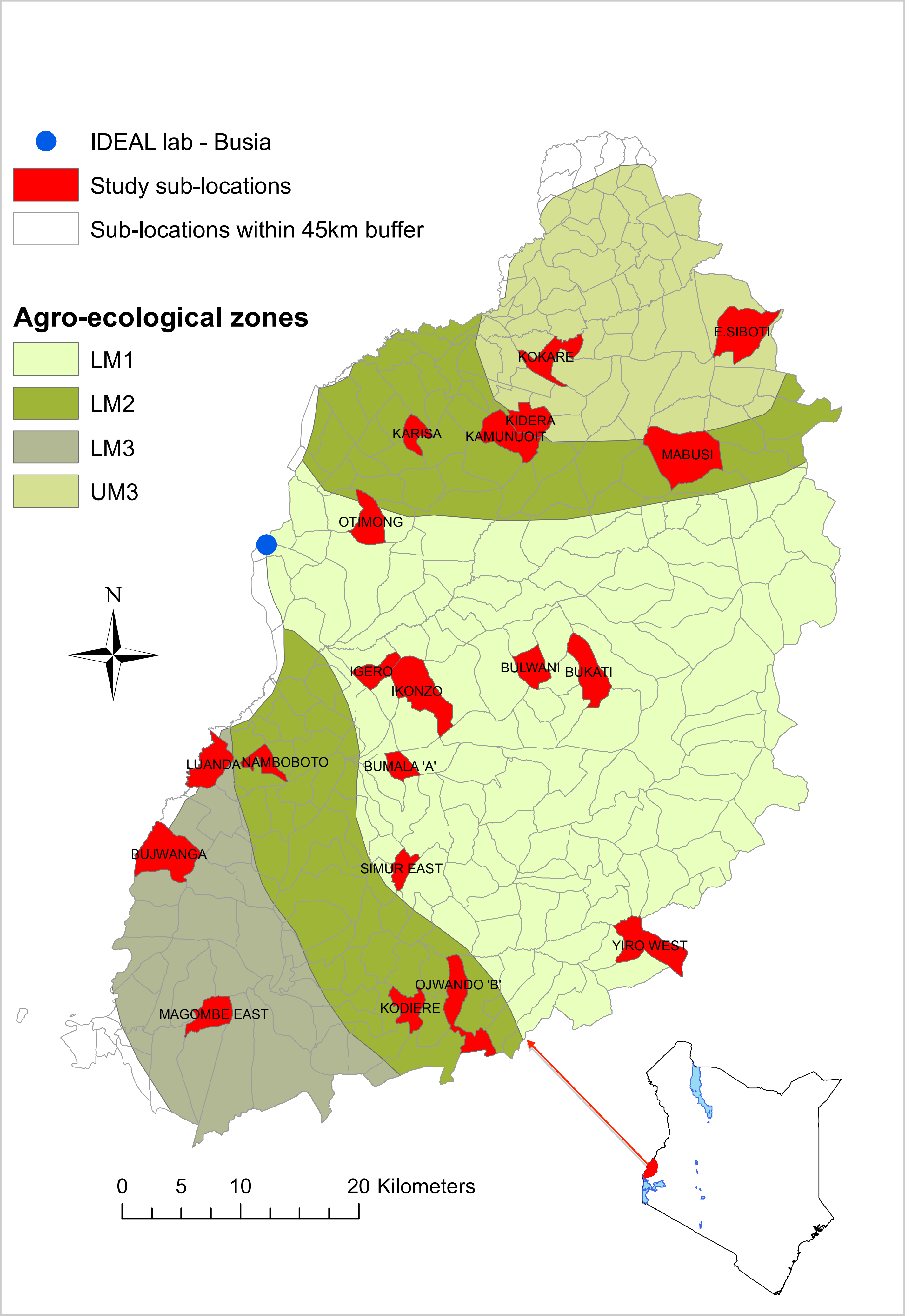
IDEAL lab - Busia
Study sub-locations
Sub-locations within 45km buffer
Agro-ecological zones
LM1
LM2
LM3
UM3
N
E.SIBOTI
KOKARE
KIDERA
KARISA
KAMUNUOIT
MABUSI
OTIMONG
IGERO
IKONZO
BULWANI
BUKATI
LUANDA
NAMBOBOTO
BUMALA 'A'
BUJWANGA
SIMUR EAST
YIRO WEST
OJWANDO 'B'
KODIERE
MAGOMBE EAST
0
5
10
20 Kilometers
