# Supplementary figures and images for: Genome-wide analysis reveals the ancient and recent admixture history of East African Shorthorn Zebu from Western Kenya
Source: Heredity (Edinb). 2014 Apr 16;113(4):297–305. doi: 10.1038/hdy.2014.31 (PMC4181064; doi:10.1038/hdy.2014.31)

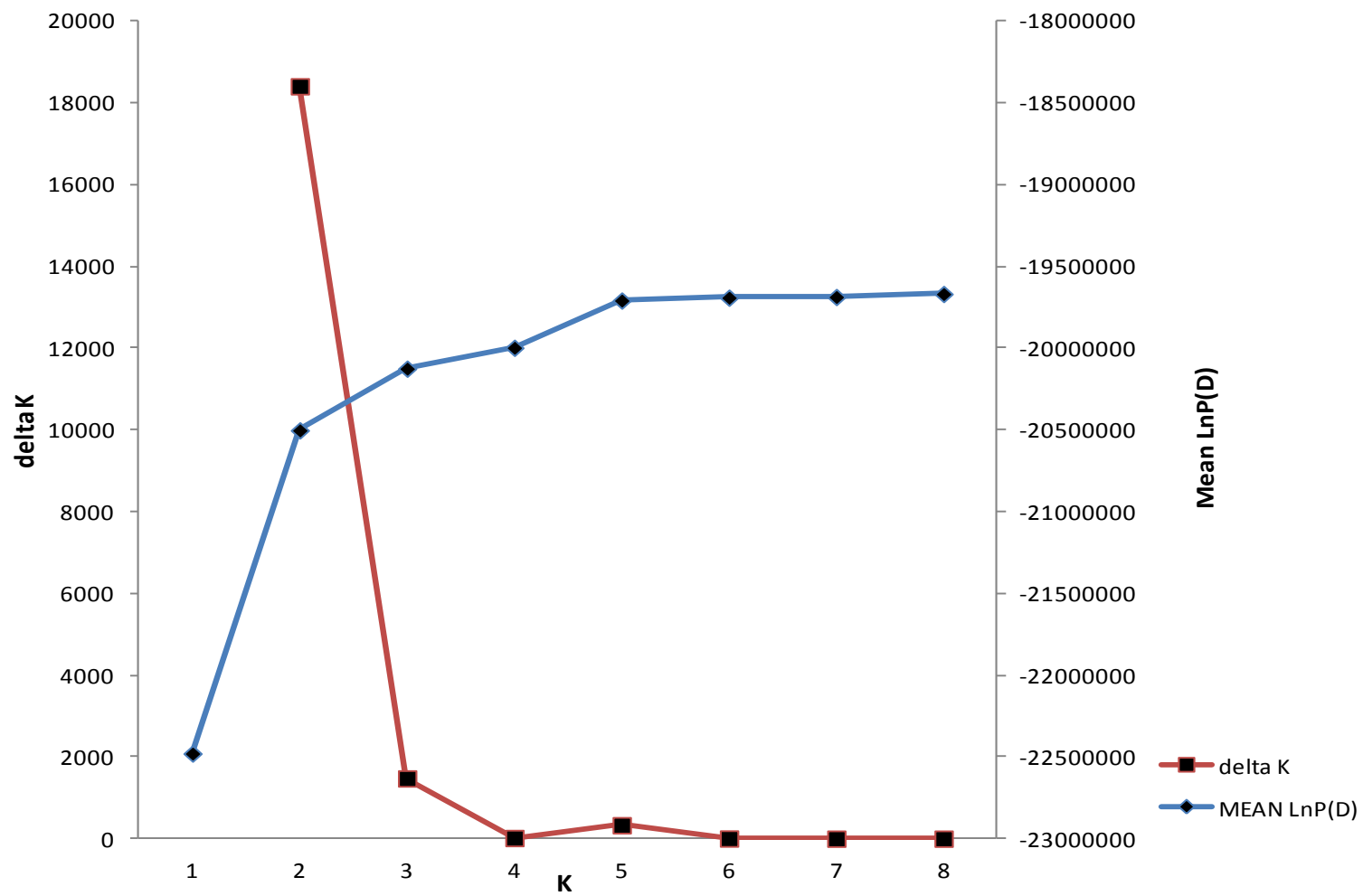

Supplement: Supplementary Figure 2 [file hdy201431x2.pdf]

East African Shorthorn Zebu

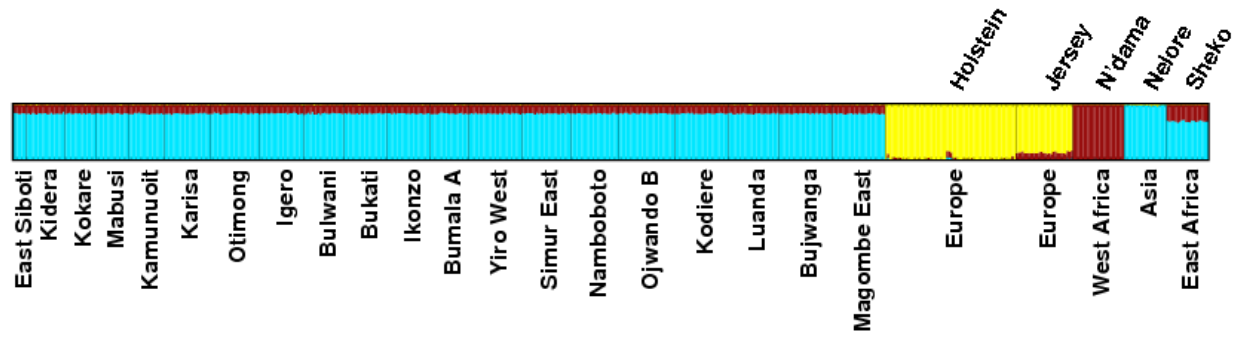

Supplement: Supplementary Figure 3 [file hdy201431x3.pdf]

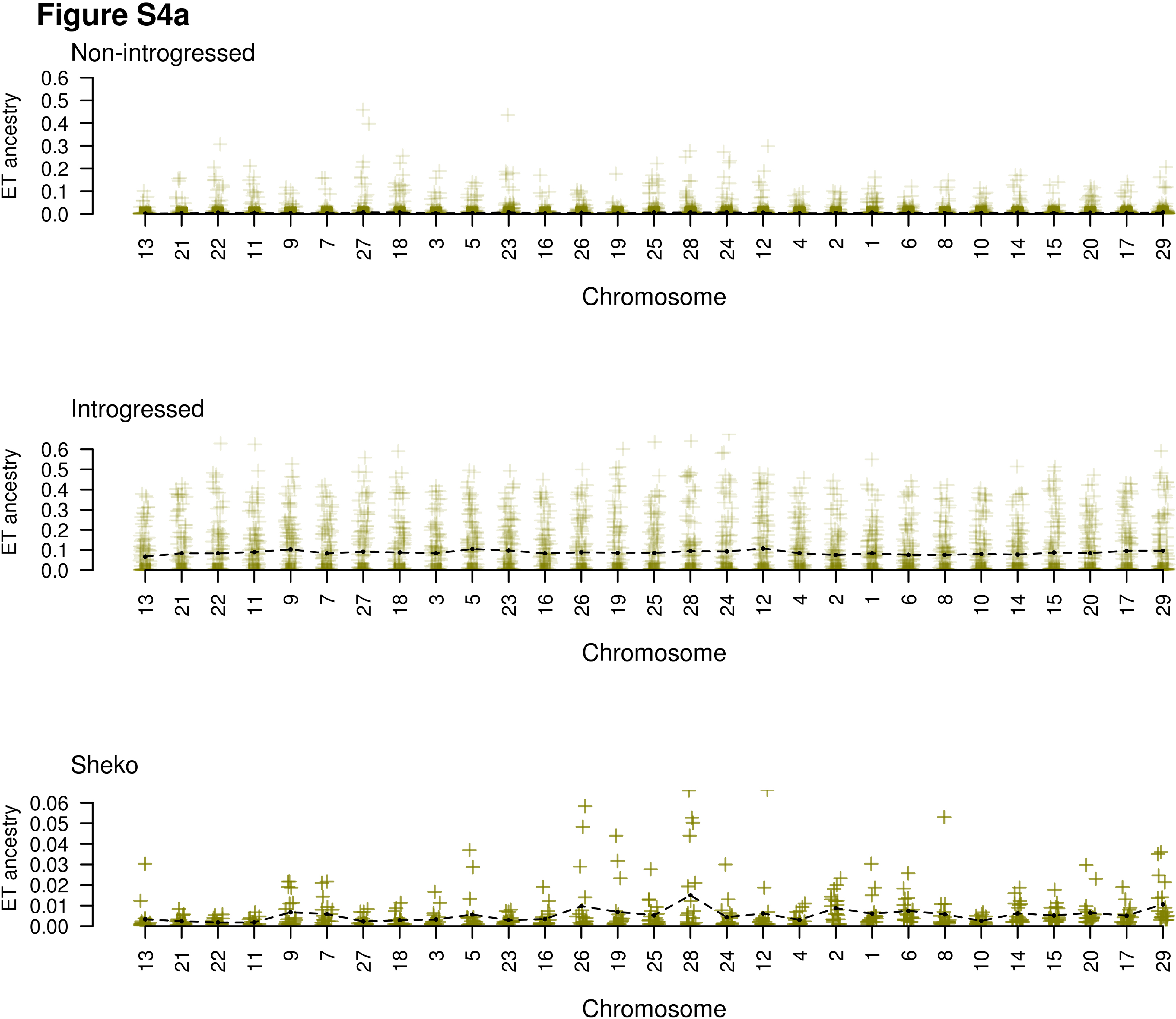

Supplement: Supplementary Figure 4a [file hdy201431x4.tif]

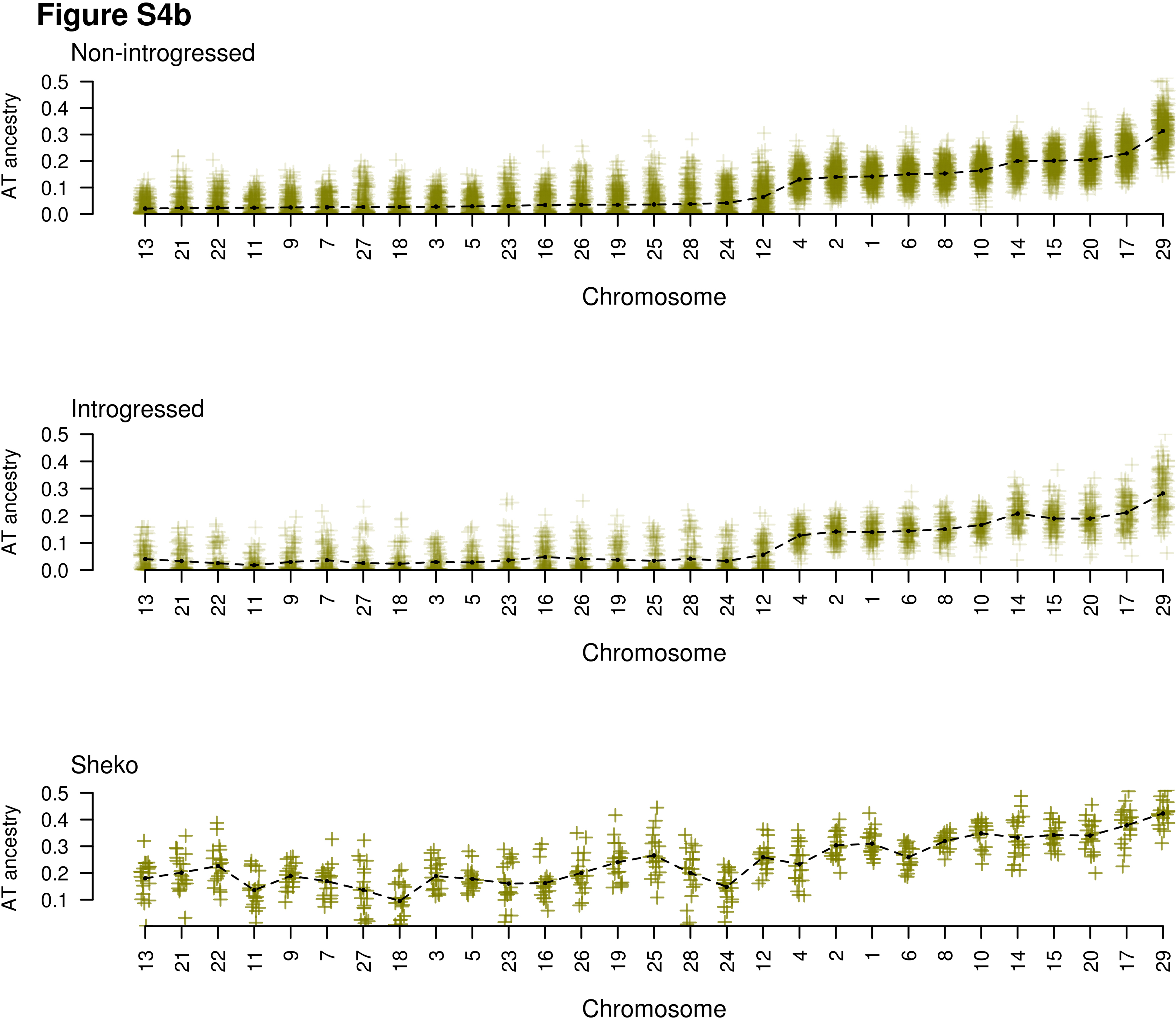

Supplement: Supplementary Figure 4b [file hdy201431x5.tif]

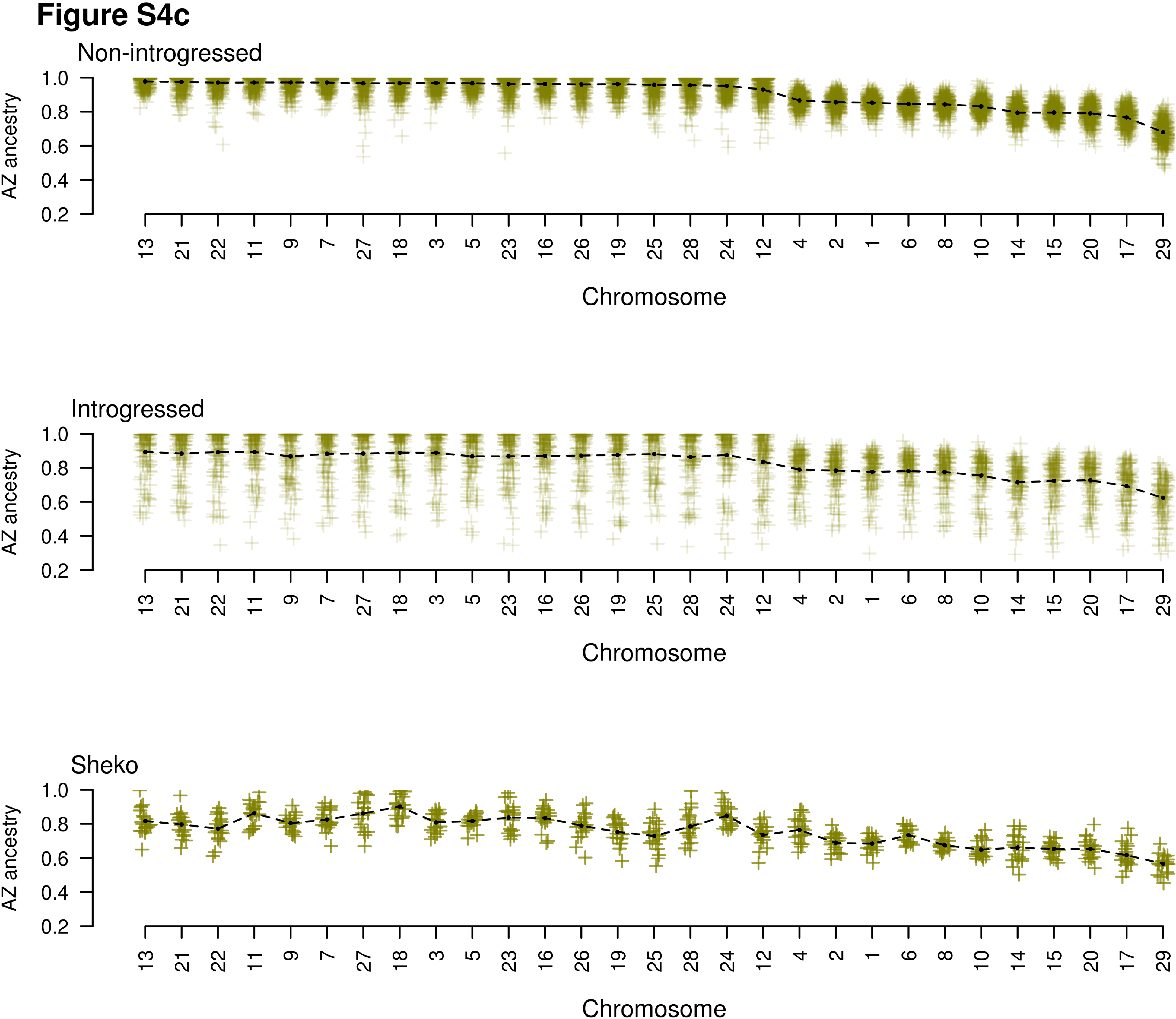

Supplement: Supplementary Figure 4c [file hdy201431x6.tif]

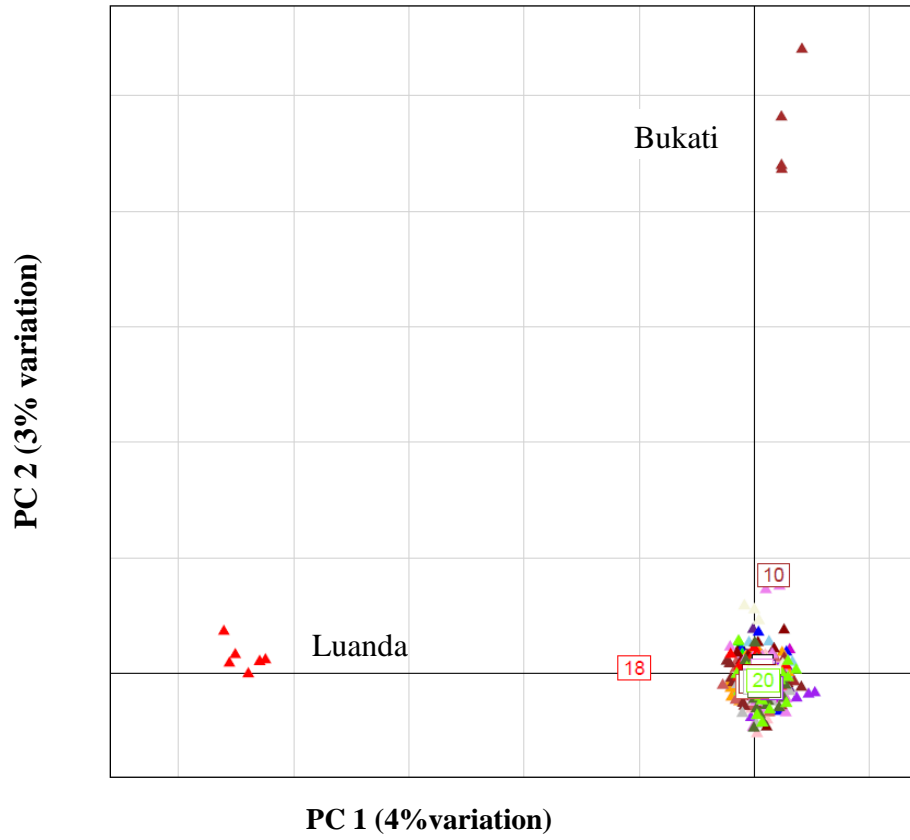

Supplement: Supplementary Figure 5a [file hdy201431x7.pdf]

East African Shorthorn Zebu

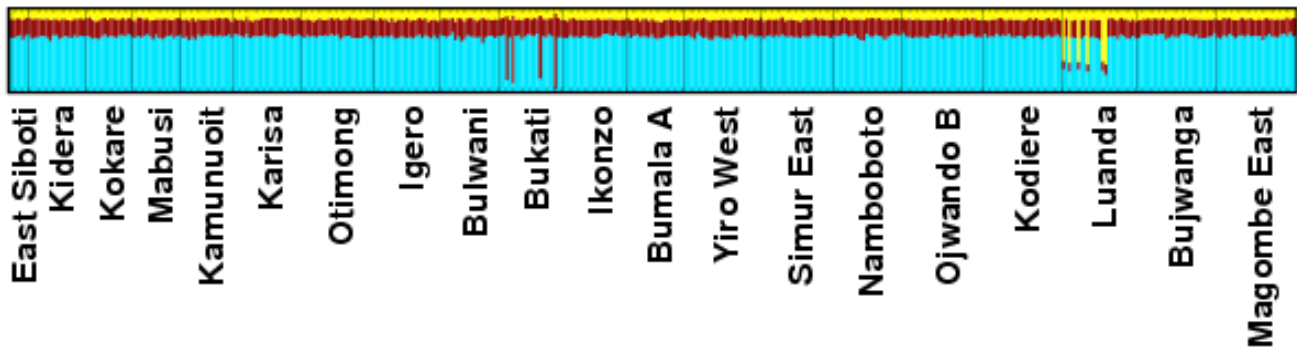

Supplement: Supplementary Figure 5b [file hdy201431x8.pdf]

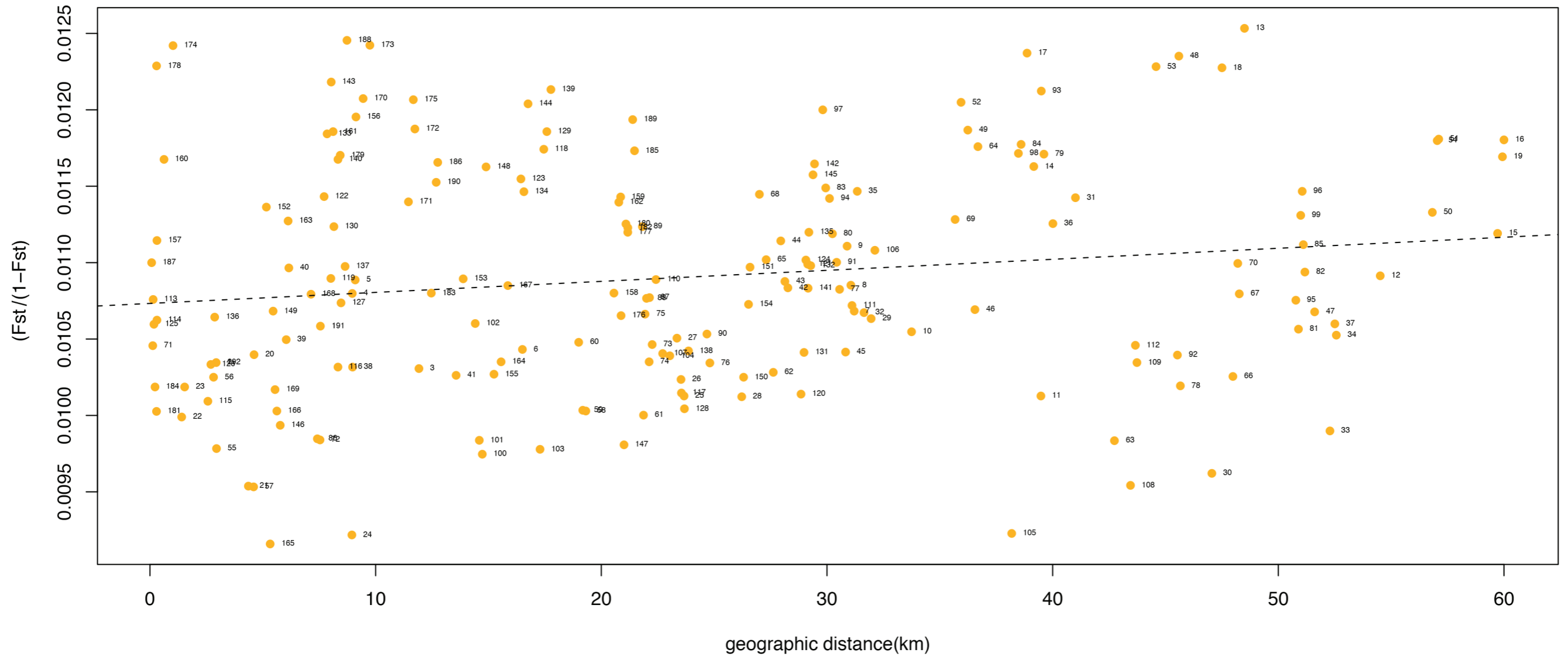

Supplement: Supplementary Figure 6 [file hdy201431x9.pdf]

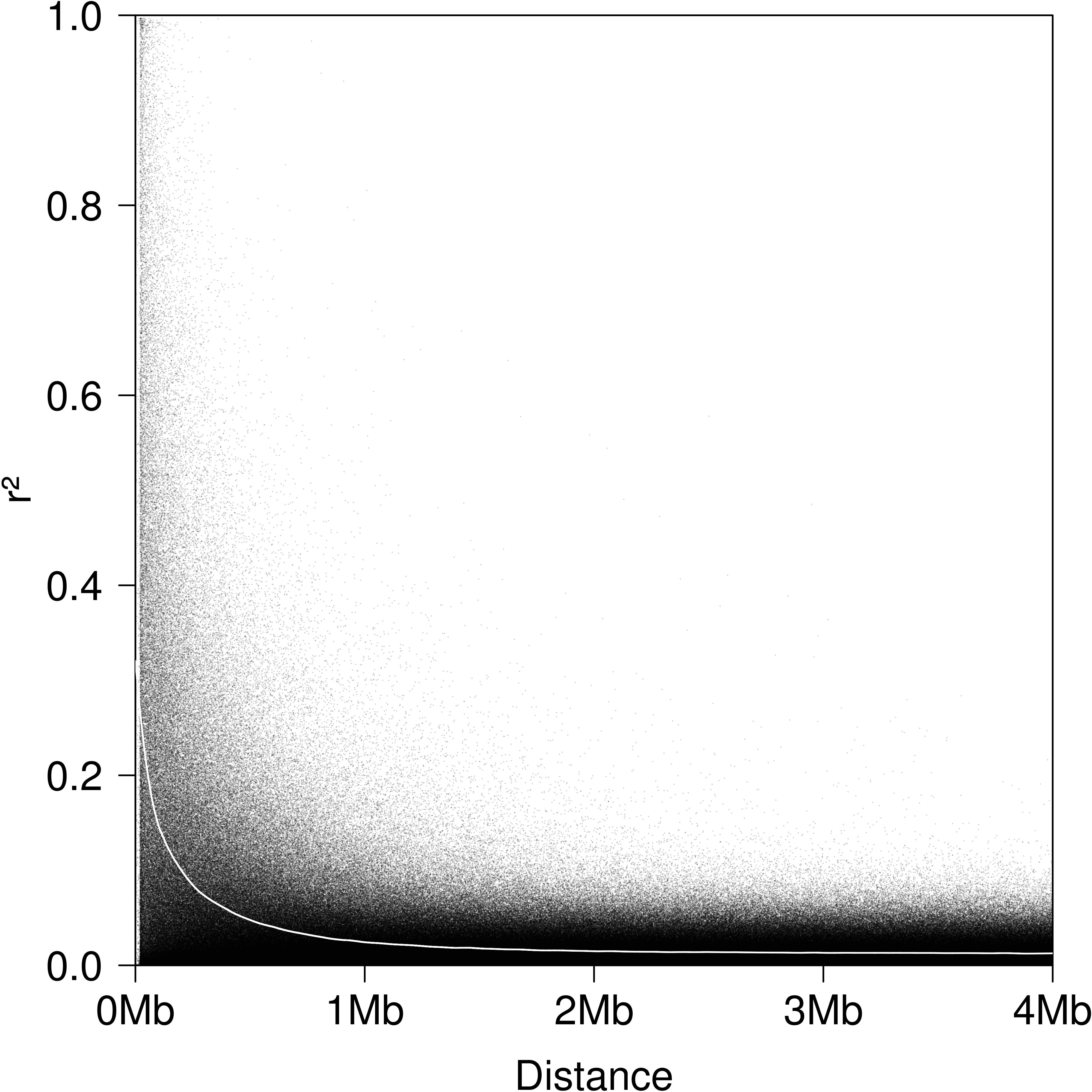

Supplement: Supplementary Figure 7 [file hdy201431x10.tif]

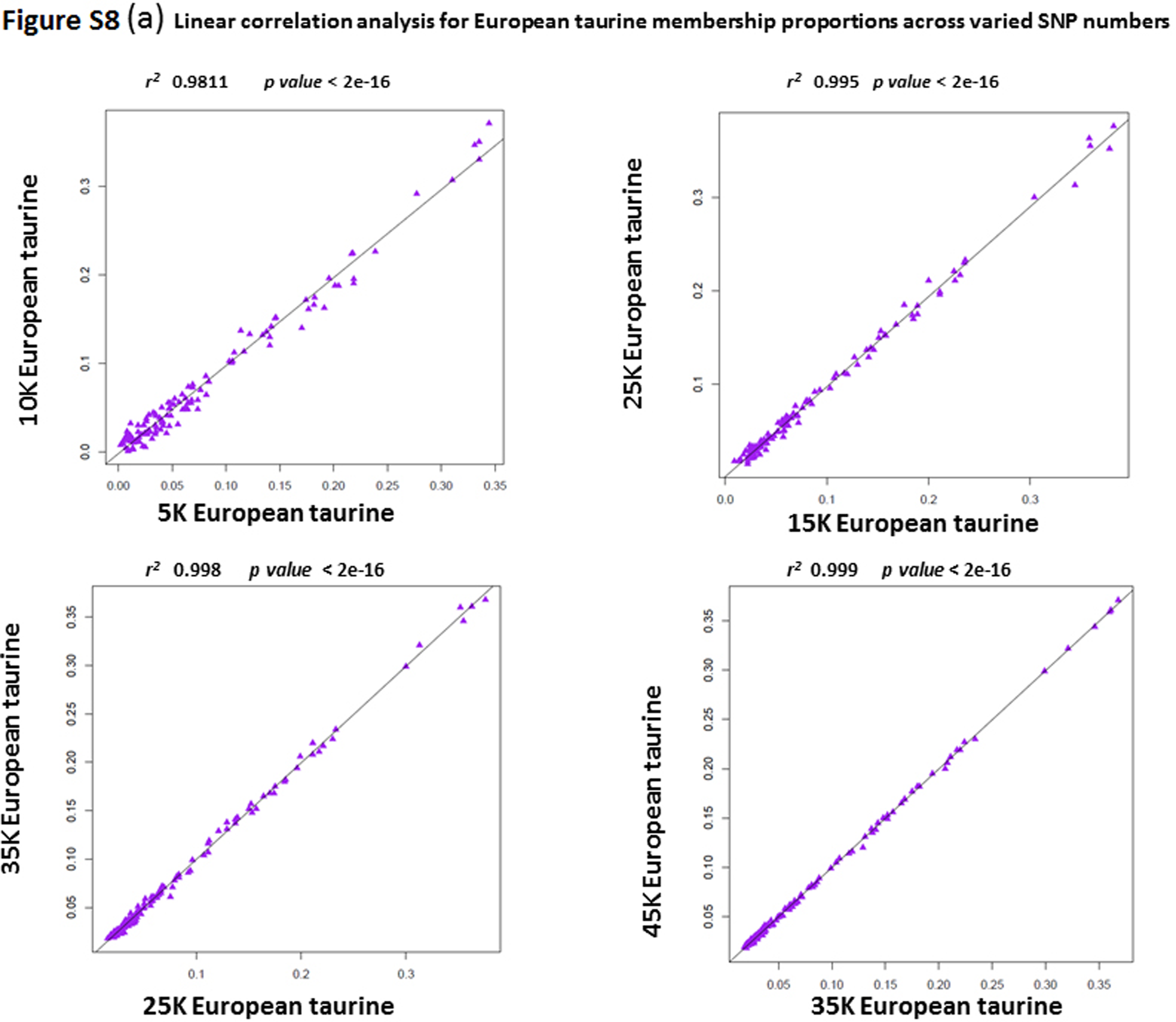

Supplement: Supplementary Figure 8a [file hdy201431x11.tif]

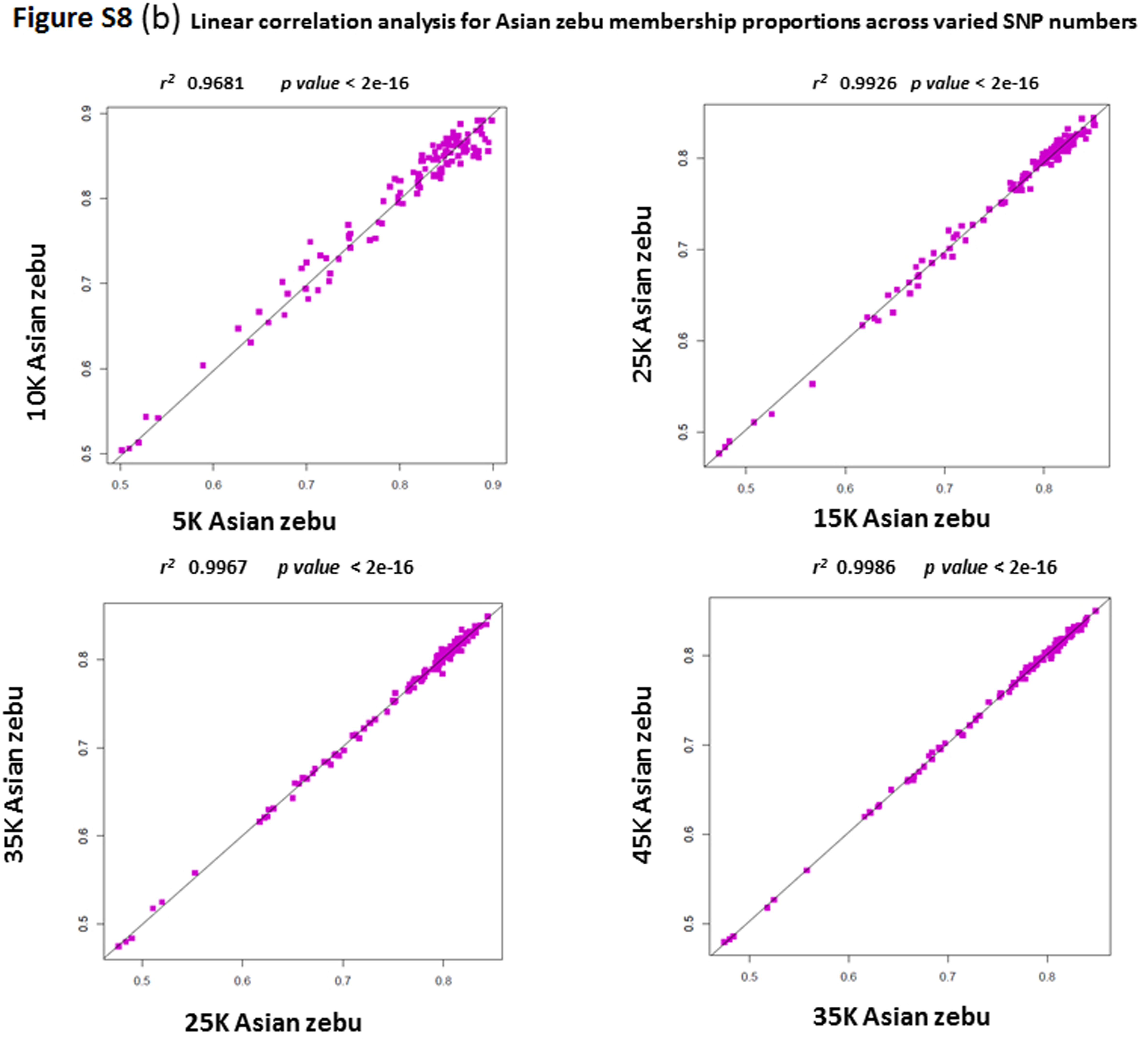

Supplement: Supplementary Figure 8b [file hdy201431x12.tif]

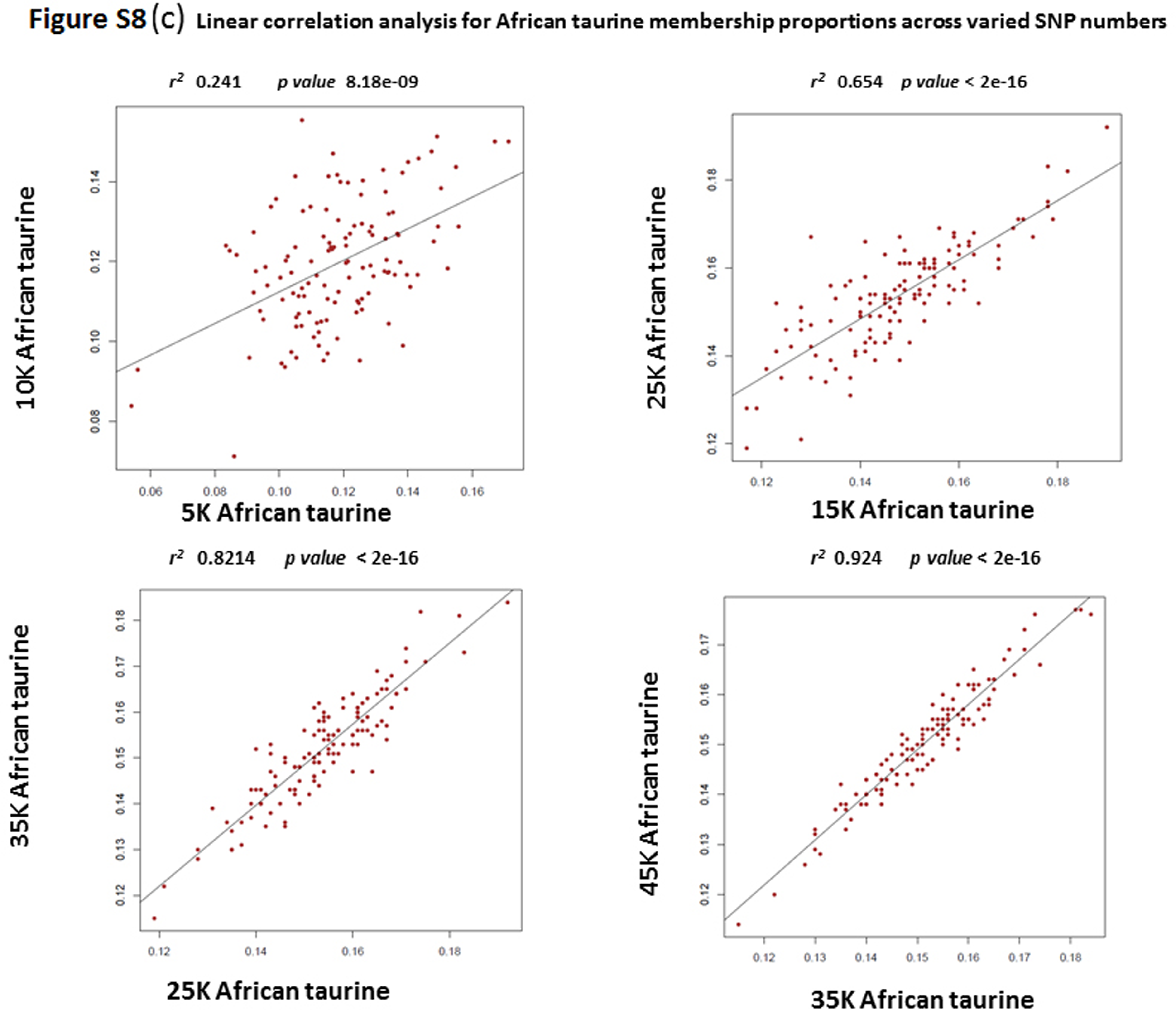

Supplement: Supplementary Figure 8c [file hdy201431x13.tif]
