# Supplementary material for: Genome-wide analysis reveals the ancient and recent admixture history of East African Shorthorn Zebu from Western Kenya
Source: Heredity (Edinb). 2014 Apr 16;113(4):297–305. doi: 10.1038/hdy.2014.31 (PMC4181064; doi:10.1038/hdy.2014.31)
Supplement: Supplementary Table S2 [file hdy201431x15.doc]

|  | **All calves**  **(EASZ n = 548)** | **Moderate and substantial calves**  **(EASZ n = 123)** | | **Non-introgressed calves**  **(EASZ n = 425)** | | **Sheko (n = 20)** |
| --- | --- | --- | --- | --- | --- | --- |
| **Between Chromosomes** |  |  |  | |  | |
| **ET** | 0.002358 ** | 0.1357^ns^ | 4.558e-07 *** | | 0.1567^ns^ | |
| **AT** | < 2e-16 *** | 4.716e-07 *** | 4.695e-12 *** | | 0.9438^ns^ | |
| **AZ** | 1.771e-09 *** | 0.02293 * | 6.425e-09 *** | | 0.9638^ns^ | |
|  |  |  |  | |  | |
| **Between calves** |  |  |  | |  | |
| **ET** | < 2.2e-16 *** | < 2e-16 *** | - | | 1 | |
| **AT** | 1 | 1 | 1 | | 1 | |
| **AZ** | < 2e-16 *** | < 2e-16 *** | 1 | | 1 | |

****P* value < 0.0001 ***P* value < 0.001

**Table S2:** Anova analysis of difference in ancestral proportion between chromosomes and calves in East African Shorthorn Zebu (EASZ) and Sheko. (Genomic ancestries: AT = African taurine, AZ = Asian zebu, ET = European taurine)
